# Porcelain Work

**Published:** 1894-11

**Authors:** J. H. Downie

**Affiliations:** Detroit, Michigan


### Porcelain Work.

BY J. H. DOWNIE, D.D.S., DETROIT, MICHIGAN.

Read before the Northern Ohio Dental Society, June, 1894.

The working of porcelain, in form in continuous gum plates, has been known to the profession for a great many years, although it has never come into general use. The working of it, by the dentist, however, in the form of crowns, bridges and inlays, is of quite recent date. There is no class of work which interests the profession more at the present day, and it is, without doubt, the coming work in dentistry. The manner in which it has been received, and the grand success which numbers have had with it, are good evidences of this. To be sure, some have failed to make a success of it, and have already thrown it aside; but their lack of knowledge of how, properly, to do the work, in no way condemns it. Because hundreds of dentists put in poor gold fillings is no reason why we should consider that a failure, which has served so grand and noble a purpose. The days when glaring gold is admired will soon cease to be, and the truly artistic operator will endeavor to imitate nature, and replace the lost organs and portions of them with a substance which matches so nearly in color that it can scarcely be detected.

Porcelain crown-work has been practiced for quite a number of years, and, when properly done, is one of the most satisfactory operations in dentistry, to both the patient and the operator. The porcelain crown has come from the old-fashioned pivot tooth, set with a wooden peg, down through various stages, each one being devised to overcome some difficulty with the former. This has led to the devising of the crown fused to a metallic cap, previously fitted to the root, and backed up with porcelain; this is the crown with which we have to deal in this paper. With it we get a perfect adaptation to the root by means of the platinum cap, which prevents the splitting of the roots, prevents the joint from decay, and the washing out of the cement, and strengthens the attachment of the crown. The anterior portion of the band is covered with porcelain of the same color as the facing, so there is no metal showing, and no necessity for cutting off the root far above the gingival margin, and lacerating the gums. It is made with the teeth used for other work, so that no special tooth is required. The backing is fused on to the facing, making a perfect union, so it is not necessary to protect the point with metal, to prevent the facing being broken off. The facing of a Richmond crown being always sure to break off, if not protected at the point with the metal backing, is no reason to suppose that a solid porcelain crown is not sufficiently strong. In the case of the metal-backed crown there is no union between the backing and the facing, the porcelain front being held in position by the two small pins alone, which certainly makes a very weak attachment. By the porcelain process, the backing is fused on to the facing, forming a perfect union, which makes, in the finished case, a solid porcelain crown.

The Logan crown, which has served so good a purpose in the past, is sufficient evidence of the strength of a solid porcelain crown. It cannot be truthfully said of it, that the porcelain is not sufficiently strong, as we have very rarely seen one broken. The porcelain is not the weak point in the Logan crown; the difficulties with it are, getting the proper adaptation, liability of the post to break and the root to split. These may be, in a measure, overcome by banding, but in doing this we present to view a strip of gold across the base of the crown, which at once puts upon it the artificial stamp.

The numerous ways in which the baking of the crown may be modified to fit the special case, and the means which we have at hand for its construction, are great advantages in the porcelain process. A crown may be made from almost any plain tooth, for either metal or rubber-work, or from almost any tooth crown manufactured. The preferable tooth for this kind of work is the flat back tooth, such as is used for bridge-work. The root should be ground off flush with the gums, the tissues being pressed away to allow of its being dressed down as far as possible without lacerating them. As the root enlarges at this point, leaving, when ground off, an overhanging edge, it is necessary to dress it off around its circumference in order to properly fit a cap to it. This can best be done with metallic disks and sharp scalers. The measurement of the root is taken with a small wire, using a small dentimeter to handle it. The wire, when removed, should be cut on the opposite side from the twist and straightened out. Laying this on a piece of platinum (30 gauge), a mark is made to show the exact length. The band is cut a little longer than marked, which allows, when the ends are beveled, for the making of a lap joint.

The soldering, as in all cases of porcelain work, is done with pure gold. The band is fitted to the root, and a cap soldered over the top, the cap being perforated to correspond with the root-canal. The post may be made from almost any kind of platinum wire, but the most suitable material is square iridio-platinum. The end of the wire is flattened with a hammer, a little broader than the space between the pins of the tooth, and a notch filed in each side so that it may be placed between the pins, which are then bent over it. This holds it sufficiently firm without soldering. The tooth or facing with post attached may now be set on in position, and waxed in place on the cap, after which it is removed and the cap filled with an investment of equal parts of pulverized silex and plaster. This when set holds the cap in position to the post, after the wax has been boiled out. The case is now ready for the adding of the porcelain. This material is mixed to a paste with water, and applied with a sable brush. It is built around the anterior portion of the band, and well out on the cap, but not over the pins of the tooth. After the first fusing these pins are filed or ground away, and in the case of a very close bite the facing may be ground away at the point of contact. More body is now added, to give the crown the desired contour, which, when fused, completes the case.

Porcelain bridge-work has much to commend it to the profession. It has no joints to absorb the secretions, and certainly merits the name of sanitary bridge-work. The unsightly gold may be dispensed with in all places except where it is necessary to make an attachment to a gold cap, and with this exception the natural appearance can be almost perfectly restored. No gold cusps or cutting edges are required to protect the bridge teeth, for precisely the same reason that they are not needed in the crown work. The crown already described is used as a support for these bridges, the only difference in its construction being that when intended for bridge-work, after setting it up in wax, it is invested, and the cap and pins soldered. This allows the crown to be set in position without the porcelain backing, and an impression to be taken with modeling compound. A model is run, the bridge teeth are waxed in, and the case invested. After removing the wax, a bar of iridio-platinum is fitted from the posts of the supporting teeth across under the pins of the bridge teeth and soldered in position. The case may then be removed from the investment and the porcelain packing added and fused on. When it is necessary to use a gold cap for the support of a bridge, it is made of an alloy of gold and platina (22 k. gold). This admits of being soldered with pure gold, is quite hard, so that its wearing qualities are excellent, and goes through the heat of the furnace without oxidation. The bar is soldered to the cap, the porcelain added and fused, and the case handled in every respect as when porcelain crowns are used for supports.

Porcelain inlaying is a grand thing in certain cases, and it is much to be regretted that the present existing circumstances do not warrant its being used more extensively. The difficulty with inlaying, is that we have not a suitable cement for setting. There is no doubt that sooner or later we will have a cement suitable for this purpose, which will make this the ideal filling. The best method we know for making them is to burnish platinum foil over and into the cavity, thus forming a matrix into which the porcelain is built and fused. Body may be added and fused two or three times until the required contour is obtained. The platinum is then peeled off, and retaining points or grooves cut into the porcelain with the edges of a diamond disk, also undercuts made in the cavity, when the piece is ready for cementing in. The mistake is often made of taking too small a piece of foil with which to make the matrix. This should be large enough to hold easily in position with the thumb and finger of the left hand, while it is being burnished into the cavity with the right. This overlapping foil, by which the matrix is handled, is left on during the process of baking. There are two principal points wherein lie the secret of having a good fit with an inlay: First, in making the walls of the cavity beveled outward for a little way fron the margin, so that when the platinum is removed, the plug will fit tightly on the margin as it bevels out, and will set in the thickness of the platinum removed, thus taking up the space occupied by it and making a perfect fit at the margin.

Second, in only partially filling the matrix with body, the first time it is baked, then replacing in the cavity and burnishing down the edge again, this corrects any springing of the matrix. For labial cavities, where there is no force to be exerted which will tend to loosen the filling, they may be used with Hill's Stopping, using a warm instrument handle to press them into place. This is, perhaps, more reliable as to lasting qualities than any cement which we have at the present day.

The Jacket crown, which is a platinum cap with a porcelain facing, is in certain cases very valuable. Its use is indicated for undeveloped teeth, usually called rice or peg teeth. These may be built out, and the normal appearance fully obtained. It is also of use for decayed teeth where the pulp has receded considerably and is in a healthy condition. It is of no use for decayed teeth of normal size, if the nerve has not receded, and they set in their proper position in the arch; because they must be ground so near to the pulp in order to get the facing on without its being too prominent, that the death of that organ is almost sure to result. There is no good reason why they should ever be used in any case where the pulp is not alive.

To construct this crown: Make a deep band or ferrule of platinum to fit the tooth which has previously been ground, slightly tapering on its sides and lingual surface, and flat and receding considerably on the labial surface. The inner portion of the band, which stands up from the tooth, is clipped off, and a flat piece soldered on to make the sloping lingual surface (supposing the crown to be an incisor or cuspid), and the anterior portion of the band ground very thin, except around the gum margin, this thin anterior portion is malleted down against the tooth, using a large foot plugger, and folding down the upper corners when necessary. A tooth is selected, either a flat back, or a plain tooth for rubber-work. It is ground away flat at the back, tapering down to as thin an edge as possible at the base. After grinding until it will set in the proper position, the cap is removed and the facing stuck on with a little porcelain body. The piece is then set on a tray, in which is a little ground silex to keep it from shifting around while in the furnace. It should be very carefully heated up in order that the facing may not be thrown off. When cool, more porcelain is added around the edges to securely attach the facing, and after fusing, the crown is ready to be cemented in place. All these operations in porcelain are made infinitely more practical by the use of the low fusing porcelain body, which enables us to back up a tooth without etching its face, to fuse on to a bridge which is soldered to a gold cap, and to match the colors almost perfectly.

---

Dr. J. Austin Dunn of Chicago has reduced the price of his Syringe with one Iridio-Platinum needle to $1.50. The Iridio-Platinum needle is something the dentists have always wanted but could not procure, and is a step in advance. The Iridium makes the needle stiff, so that it will wear smooth and is more lasting and durable. Another advantage is, that it can be held in the flame of the spirit lamp and cleaned by burning out the debris without danger of softening it. The price for extra needles has been fixed at fifty cents, so that they are within the reach of all.

